# Buffalo Medical College—New Edifice

**Published:** 1849-12

**Authors:** 


*Buffalo Medical College—New Edifice.*—The new College edifice was formally opened, by appropriate public exercises, on the 7th ult, the day of the commencement of the term now in progress. An address was delivered by the Hon. Millard Fillmore, Vice President of the United States, and Chancellor of the University. The distinguished speaker gave a learned, and highly interesting history of the origin of Universities, and discussed the character and value of collegiate honors, concluding with an eloquent and impressive appeal to the citizens of Buffalo, in behalf of the other departments of the University, which, as yet, remain to be organized. A large and highly intelligent auditory were present on the occasion. The Chancellor was followed by an address by the Dean of the medical Faculty, embracing a historical sketch of the rise and progress of the medical College. The latter has been published entire, in the Daily Commercial Advertiser, of this city, and in the Western Literary Messenger.

After the addresses, the ladies and gentlemen assembled on the occasion, visited the several apartments of the College, and participated in a collation provided by the medical Faculty. The new college edifice is entirely completed, excepting the section to be finished as a museum. The completion of the latter is deferred, as it is not needed at present, a hall, 50 feet by 20, being appropriated for a museum, in the present arrangement, which is adequate to all the wants of the institution. We think we are not biased by a feeling of partiality, which is natural, if not excusable, when we say, that we doubt if a more commodious and convenient building for purposes of medical education exists in the United States. The lecture rooms present one peculiar feature which has excited much attention. They are seated with cast iron arm chairs, without bottoms, firmly screwed upon cushioned benches. They are pleasing to the eye, and exceedingly comfortable to the sitter, in fact, combining all the advantages of a luxurious arm chair. The right arm of each chair is expanded for the convenience of taking notes. The chair is light, weighing only 18 pounds, and, being painted, presents quite an elegant appearance. We know of no educational institution in which similar provisions for the physical ease of pupils exist. If the faculties of the mind are inactive, it will not be because the attention is absorbed by uneasy sensations of the body, and while the latter are obviated, the indulgence of postures favorable to indolence and sleep, is effectually prevented.

The class now in attendance numbers 107 which certainly argues well for the continued prosperity of the institution.

